# The shortest path problem in the stochastic networks with unstable topology

**DOI:** 10.1186/s40064-016-3180-7

**Published:** 2016-09-13

**Authors:** Gholam H. Shirdel, Mohsen Abdolhosseinzadeh

**Affiliations:** Department of Mathematics, Faculty of Basic Science, University of Qom, Qom, Iran

**Keywords:** Stochastic network, The stochastic shortest path, Discrete time Markov chain, Arrival probability, Primary 90C59, 90C35; Secondary 90C40

## Abstract

The stochastic shortest path length is defined as the arrival probability from a given source node to a given destination node in the stochastic networks. We consider the topological changes and their effects on the arrival probability in directed acyclic networks. There is a stable topology which shows the physical connections of nodes; however, the communication between nodes does not stable and that is defined as the unstable topology where arcs may be congested. A discrete time Markov chain with an absorbing state is established in the network according to the unstable topological changes. Then, the arrival probability to the destination node from the source node in the network is computed as the multi-step transition probability of the absorption in the final state of the established Markov chain. It is assumed to have some wait states, whenever there is a physical connection but it is not possible to communicate between nodes immediately. The proposed method is illustrated by different numerical examples, and the results can be used to anticipate the probable congestion along some critical arcs in the delay sensitive networks.

## Background

The deterministic shortest path problem has been studied extensively and applied in many fields of optimization; there are polynomial time algorithms to solve the deterministic shortest path problem (Dijkstra 1959; Bellman 1958; Orlin et al. 2010). However, paths in networks should be reliable to transmit flow from a source node to a destination node especially in delay sensitive networks. The best connection helps to avoid traffic congestion in networks. So, the arrival probability is used to evaluate the reliability of paths and it has been considered as an optimality index of the stochastic shortest path length (Bertsekas and Tsitsiklis 1991; Fan et al. 2005; Kulkarni 1986; Shirdel and Abdolhosseinzadeh 2016). The stochastic shortest path problem (SSP) is defined as the best path with stochastic optimality condition. Liu (2010) assumed the arc lengths to be uncertain variables. Pattanamekar et al. (2003) considered the individual travel time variance and the mean travel time forecasting error. Also, Hutson and Shier (2009) and Rasteiro and Anjo (2004) supposed two criteria: mean and variance of path length. Fan et al. (2005) assumed known conditional probabilities for link travel times that each link could be congested or uncongested. Wu et al. (2004) modeled a stochastic and time-dependent network with discrete probability distributed arc weights. Peer and Sharma (2007) assumed two kinds of nodes, possible failure and always working. Ji (2005) solved three models of the shortest path by integrating stochastic simulation and genetic algorithm. The considered model in this paper is a directed acyclic stochastic network with known discrete distribution probabilities of leaving or waiting in nodes.

Our criterion to evaluation of the connections from the source node toward the destination node in the network is presented as the arrival probability, which is obtained by the established discrete time Markov chain (DTMC) in the network (Shirdel and Abdolhosseinzadeh 2016); then, the best possible connection is determined with the largest arrival probability. Liu (2010) converted his models into deterministic programming problems. Hutson and Shier (2009) and Rasteiro and Anjo (2004) obtained the maximum expected value of a utility function. Fan et al. (2005) applied a procedure for dynamic routing policies. Nie and Fan (2006) formulated the stochastic on-time arrival problem with dynamic programming, and Fan et al. (2005) minimized the expected travel time.

In this paper, the maximum arrival probability from a given source node to a given destination node is computed according to known discrete distribution probabilities of leaving or waiting in nodes, and a DTMC stochastic process is used to model the problem rather than dynamic programming or stochastic programming. Kulkarni (1986) developed a method based on a continuous time Markov chain (CTMC) to compute the distribution function of the shortest path length. Azaron and Modarres (2005) applied Kulkarni’s method to queuing networks. Thomas and White (2007) modeled the problem of constructing a minimum expected total cost route as a Markov decision process. They wanted to respond to dissipated congestion over time according to some known probability distribution.

The arrival probability gives overall information of the network conditions to transmit flow from the source node toward the destination node. Two conditions at any state of the established DTMC are assumed: departing from the current state to a new state, or waiting in the current state with expecting better conditions. There are several unstable connections between nodes. The leaving distribution probability from one node toward another node is known as the probability that their connected arc is uncongested. A DTMC with an absorbing state is established and the transition matrix is obtained. Then, the arrival probability from the source node toward the destination node is computed as the multi-step transition probability from the initial state to the absorbing state in DTMC. The arrival probability introduced by Shirdel and Abdolhosseinzadeh (2016) is reviewed in this paper, and it is extended and the concepts and definitions are organized to find the stochastic shortest path.

This paper is organized as follow. In “The unstable topology of the network” section some definitions and assumptions of networks with unstable topology is introduced. The concept of the stochastic process and the established DTMC in the network is described in “The established discrete time Markov chain” section; also, the computations of the arrival probability and the stochastic shortest path are presented in “The established discrete time Markov chain” section. “Numerical results” section contains some numerical results of implementation of the proposed method on some networks with various topologies.

## The unstable topology of the network

In this section, we introduce some definitions and assumptions of networks with unstable topology. Let network $G=(N,A)$, with node set *N* and arc set *A*, be a directed acyclic network. Then, we can label its nodes in a topological order such that for any $(i,j)\in A, i<j$ (Ahuja et al. 1993). The physical topology for any $(i,j)\in A$ shows the possibility of communication between nodes $i,j\in N$ in the network. In the transportation networks there are some physical connections between nodes, but we cannot traverse anymore toward the destination node because of probable congestion. If there are some facilities in the network *G*, but it is not possible to use them continuously, then *G* has unstable topology. So, for any arc $(i,j)\in A$ it is not mean there is a stable communication between nodes $i,j\in N$ all the time (it could be probably congested). For any node *i*, it is supposed that the uniform distribution probabilities of leaving arcs (*i*, *j*) to be uncongested are known (Shirdel and Abdolhosseinzadeh 2016).

**Fig. 1:**
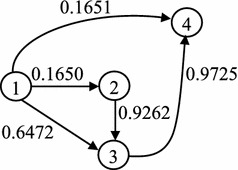
The example network with 4 nodes and 5 arcs

Now, consider the situation that some arcs are congested and flow cannot leave because of the unstable topology. There are two kinds of wait situations: first, waiting in a particular node with expecting some facilities to release from the current condition, and it is called option 1; second, traversing some arcs those do not lead to visit a new node, and it is called option 2. For example, if it is decided to be in node 3 in the example network (Fig. 1), arc (1, 3) does not cause to visit a new node whereas arc (3, 4) leads to the new node 4. The produced wait situations are more extended than queuing networks considered by Azaron and Modarres (2005) and Thomas and White (2007).

The stochastic variable of arc (*i*, *j*) according to the unstable topology is shown by $x_{ij}$. If $x_{ij}=1$, it is possible to traverse arc (*i*, *j*), and otherwise $x_{ij}=0$. The probability that arc (*i*, *j*) to be uncongested is $q_{ij}=Pr[x_{ij}=1]$, and it represents the uniform probability that node *i* is leaved toward node *j* (an adjacency node). Then, the wait probability in node *i*, is $q_{ii}=1-\sum _{\{j:(i,j)\in A\}}q_{ij}$, and it is the probability that leaving arcs by node *i* are congested.

Figure 1 shows the example network with its topological ordered nodes and it is the initial physical topology of the network. The numbers on arcs show the leaving probabilities $q_{ij}$. Node 1 is the source node and node 4 is the destination node. It is not possible to traverse arc (2, 4) because it does not exist in the physical topology of the example network. However, the arcs in the physical topology could be congested according to the known distribution probabilities.

## The established discrete time Markov chain

In this section, the proposed DTMC by Shirdel and Abdolhosseinzadeh (2016) is reviewed. The discrete time stochastic process $\{X_r,r=1,2,3,\ldots \}$ is called Markov chain ($X_r$ shows the process position), if it satisfies the following Markov property (see Ross 2006 and Thomas and White 2007)

$$Pr[X_{r+1}=S_l|X_r=S_k,X_{r-1}=S_m,\ldots ,X_1=S_n]=Pr[X_{r+1}=S_l|X_r=S_k]=p_{lk}.$$

Any state $S_l$ of the established DTMC determines the traversed nodes of the original network. For the example network (Fig. 1) the created states $S_i$, are shown in Table 1. The conditional probability of the next state depends on the current state and independent of the previous states. Let $S=\{S_i,i=1,2,3,\ldots \}$, the initial state $S_1=\{1\}$ of DTMC contains the single source node and the absorbing state $S_{|S|}=\{1,2,\ldots ,|N|\}$ contains all nodes of the network and it is not possible to depart; so, *S* is a finite state space (it is not possible to depart from $S_{|S|}$).

**Fig. 2:**
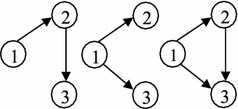
Constructed connected components of state $S_4$

For the example network, the absorbing state $S_5=\{1,2,3,4\}$ contains all nodes of the network; and the instance state $S_4$ of the state space *S* (Table 1) contains nodes $\{ 1,2,3\}$ and all connected components of the example network, those are constructed by nodes 1, 2 and 3, as seen in Fig. 2.

**Table 1:** The state space of the established DTMC for the example network

States	$S_1$	$S_2$	$S_3$	$S_4$	$S_5$
Nodes	$\{1\}$	$\{1,2\}$	$\{1,3\}$	$\{1,2,3\}$	$\{ 1,2,3,4\}$

The final state contains the destination node of the network, where DTMC does not progress anymore, and it is called assumption i. The states of the established DTMC contain the traversed nodes of the network, those are reached from some nodes in a previous state, and it is called assumption ii. It is not allowed to return from the last traversed node; however, it is possible to wait in the current state. Clearly, a new state is revealed if a leaving arc $(i,j)\in A$ is traversed such that the current node *i* is contained in the current state and the new node *j* is contained in the new state, and it is called assumption iii. As previously said, the wait states are one of option 1 or option 2.

**Fig. 3:**
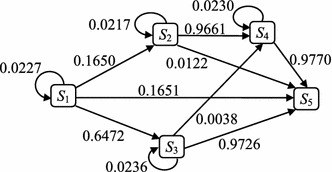
The state space diagram of the established DTMC

The state space diagram of the established DTMC for the example network is constructed as Fig. 3; the values on arcs show the wait and the transition probabilities.

### The transition and the wait probabilities

The transition probabilities $p_{kl}$ satisfy the following conditions

- $$0\le p_{kl}\le 1$$ for $$k=1,2,\ldots ,|S|$$ and $$l=1,2,\ldots ,|S|$$
- $$\sum _l{p_{kl}}=1$$, for $$k=1,2,\ldots ,|S|$$.

The transition probabilities are elements of matrix $P_{|S|\times |S|}$, where $p_{kl}$ is *k*th row and *l*th column of matrix *P*, and it is called the transition matrix or Markov matrix (Ibe 2009). The following theorems are used to obtain the transition matrix of the established DTMC in the network by Shirdel and Abdolhosseinzadeh (2016). The transition probabilities (except the absorbing state) are obtained by Theorem 1.

#### **Theorem 1**

*If*$p_{kl}$* isklth element of matrix P, that*$k \ne l, l < |S|$* and*$S_{k}=\{v_0=1,v_1,\ldots ,v_m\}$* is the current state, then the transition probability from state*$S_{k}$*to state*$S_{l}$*is computed as follow*

*if*$l < k$*then*$p_{kl} = 0$, *otherwise if*$l > k$*then*

$$p_{kl}=Pr\left[ \bigcup _{\left( v,w\right) \in \Psi }{E_{vw}}\right] \times \left( \prod _{\left( v,w\right) \in \Psi }{\left( 1-\sum _{\begin{array}{c} \left( v,u\right) \in A\\ u\ne w,u\notin S_k \end{array}}{q_{vu}}\right) }\right) \times q_{v_mv_m}+q_{v_mw}.$$

$E_{vw}$*denotes the event which arc* (*v*, *w*) *is traversed during transition from*$S_k$*to*$S_l$*and*$\Psi =\{(v,w)\in A:v\in S_k\backslash \{v_m\},w\in S_{l}\backslash S_k,|S_l\backslash S_k|=1\}$.

#### Proof

Since, it is not allowed to traverse from one state to the previous states (assumption ii), then necessarily $p_{kl}=0$, for $l\ <\ k$. Otherwise, suppose $l\ >\ k$, during transition from the current state $S_k$ to the new state $S_l$, it should be reached just one node other than the nodes of the current state, so $|S_l\backslash S_k|=1, v\in S_k$, and $w\in S_l\backslash S_k$ are held by assumption ii and iii. Two components of $p_{kl}$ formula should be computed.

In the last node $v_m$ of the current state $S_k$, it is possible to wait in $v_m$ with probability $q_{v_mv_m}$. Notice, it is not possible to wait in the other nodes $v\in S_k\backslash \{v_m\}$ because it should be leaved to construct the current state, however it is not necessary for node $v_m$ with the largest label (leaving $v_m$ leads to a new node, and therefore results in a new state). If $w\in S_l\backslash S_k$, then one or all of events $E_{vw}$ (i.e. to traverse a connecting arc between a node of the current state and another node of the new state) can happen for $\left( v,w\right) \in \Psi$, and the arrival probability of node $w \in S_l$ from the current state $S_k$ is equal to $Pr[\bigcup _{\left( v,w\right) \in \Psi }{E_{vw}}]$. The collection probability should be computed because of deferent representations of the new state (for example see Fig. 2). Then, the nodes of the current state $v\in S_k\backslash \{v_m\}$ (while waiting in $v_m$) should be prevented from reaching other nodes $u\notin S_k$ and $u\ne w$ (assumption iii), so arcs $\left( v,u\right)$ are not allowed to traverse and they are excluded simultaneously, thus it is equal to $$\prod _{\left( v,w\right) \in \Psi }{(1-\sum _{\begin{array}{c} \left( v,u\right) \in A \\ u\ne w,u\notin S_k \end{array}}{q_{vu}))}}$$. The other possibility in node $v_m$, that is leaving it toward the new node $w\in S_l\backslash S_k$ with probability $q_{v_mw}$. $\square$

**Fig. 4:**
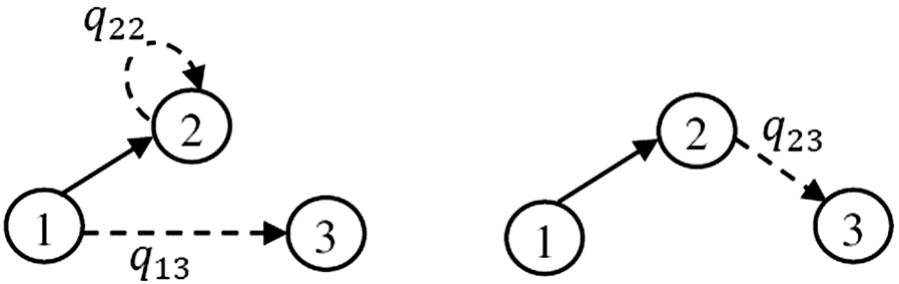
The constructed states during transition from $S_2$ to $S_4$

For example, in the established DTMC of the example network, the transition probability $p_{24}$ is computed by the constructed components as shown in Fig. 4; and it is $P(E_{13})\times (1-q_{14})\times q_{22}+q_{23}$, where $P(E_{13})= q_{13}$, then $p_{24}=q_{22}\times q_{13}\times (1-q_{14})+q_{23}$. It is possible to wait in node 2 but not other nodes of the current state $S_2=\{1,2\}$; where, by traversing arc (1, 3) or (2, 3) the new state $S_4=\{1,2,3\}$ is revealed.

Theorem 2 describes the transition probabilities to the absorbing state $S_{|S|}$, and they are the last column of the transition matrix *P*.

#### **Theorem 2**

*To compute the transition probability from state*$S_k=\{v_0=1,v_1,\ldots ,v_m\}$*to the absorbing state*$S_{|S|}$*for*$k=1,2,\ldots ,|S|-1$, *which isk*|*S*|*th element of matrixP*, *suppose*$v_n\in S_{|S|}$*is the given destination node of the network then*

$$p_{k|S|}=Pr\left[ \bigcup _{v\in S_k,(v,v_n)\in A}E_{vv_n}\right].$$

$E_{vv_n}$*denotes the event that arc*$(v,v_n)\in N$*of the network is traversed during the transition from*$S_k$*to*$S_{|S|}$.

#### Proof

To compute the transition probabilities $p_{k|S|}$, for $k=1,2,\ldots ,|S|-1$ it should be noticed the final state is the absorbing state $S_{|S|}=\{1,2,3,\ldots ,|N|\}$ containing all nodes of the network, and the stochastic process does not progress any more (assumption i). So, it is sufficient to consider leaving arcs $(v,v_n)$ from $v\in S_k$, the nodes of the current state, toward the destination node $v_n\in S_{|S|}$. Then, one or all of events $E_{vv_n}$ (i.e. to traverse a connecting arc between a node of the current state and the destination node of the absorbing state) can happen and the transition probability from the current state $S_k$ to the absorbing state $S_{|S|}$ is totally equal to $Pr[\bigcup _{v\in S_k,(v,v_n)\in A}E_{vv_n}]$. The collection probability should be computed because of different representations of the states (for example see Fig. 2). $\square$

For state $S_4$, transition probability $p_{45}$ is obtained by $P(E_{14}\cup E_{34}\cup E_{24})$, however $q_{24}=0$ as seen in Fig. 1, then $p_{45}=q_{14}+q_{34}-q_{14}\times q_{34}$. The wait probabilities, those are the diagonal elements of the transition matrix *P*, are obtained by Theorem 3.

#### **Theorem 3**

*Suppose*$S_k=\{v_0=1,v_1,\ldots ,v_m\}$*is the current state, then the wait probability*$p_{kk}$*iskkth element of matrixP and it is*

$$\begin{aligned} p_{kk}= {\left\{ \begin{array}{ll} 1-\sum ^{|S|}_{j=k+1}p_{kj} &\quad \text {if } k<|S|\\ 1 &\quad \text {if }k=|S|. \end{array}\right. } \end{aligned}$$

#### Proof

The wait probabilities $p_{kk}$ are the complement probabilities of the transition probabilities from the current state $S_k$, for $k=1,2,\ldots ,|S|-1$, toward the all departure states $S_j$, for $j=k+1,k+2,\ldots ,|S|$. Then, we have $p_{kk}=1-\sum ^{|S|}_{j=k+1}p_{kj}$, for $k=1,2,\ldots ,|S|-1$, in other word, they are the diagonal elements of matrix *P*, those are computed for any row $k=1,2,\ldots ,|S|-1$ of the transition matrix (see Ibe 2009). The absorbing state $S_{|S|}$ does not have any departure state, so $p_{|S||S|}=1$ as the transition matrix *P*. $\square$

### The arrival probability

The arrival probability determines the overall reliability of connections in the network, and it shows the probability that they are not congested during the transmission of flow from the source node to the destination node in the network. The arrival probability is defined as multi-step transition probability from the initial state $S_1$ to the absorbing state $S_{|S|}$ in the established DTMC. According to the assumptions i, ii and iii, the state space of DTMC is directed and acyclic (otherwise return to the previous states is allowed contradictively). Out-degree of any state is at least one (without loop wait transition arcs consideration), except the absorbing state $S_{|S|}$, then for any state $S_k$, there is one\multi-step transition from the initial state to the absorbing state that traverses state $S_k$. Consequently, the absorbing state is accessible from the initial state after some finite transitions. Let $p_{kl}(r)=Pr[X_{m+r}=S_l|X_m=S_k]$ denote the conditional probability that the process will be in state $S_l$ after exactly *r* transitions, given that it is presently in state $S_k$. So, if matrix *P*(*r*) is the transition matrix after exactly *r* transitions, it can be shown that $P(r)=P^r$, and let $p_{kl}(r)$ be *kl*th element in matrix $P^r$ (see Ibe 2009). Thus, the arrival probability after exactly *r* transitions is $p_{1|S|}(r)=Pr[X_r=S_{|S|}|X_0=S_1]$ and it is the 1|*S*|th element in the matrix $P^r$.

**Fig. 5:**
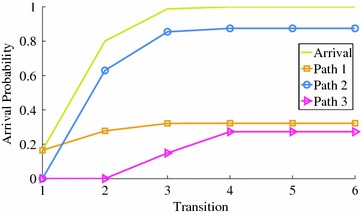
The arrival probabilities of the example network

For the example network, we want to obtain the probability of the arrival node 4 from node 1. The arrival probability $p_{15}(r)$ is obtained as shown in Fig. 5 after six transitions. For *r* sufficiently large, the probabilistic behavior of DTMC becomes independent of the starting state i.e. $Pr[X_r=S_{|S|}|X_0=S_1]=Pr[X_r=S_{|S|}]$, that is the multi-step transition probability (Ibe 2009).

### The stochastic shortest path 

Now, we extended Shirdel and Abdolhosseinzadeh (2016) method to compute the arrival probability for a specific path, it should be considered as the probable shortest path. So, it is enough to put some conditions on the leaving probabilities $q_{ij}$, those enforce the nodes of the considered shortest path to be reached sooner than the other nodes in the network. Thus, the stochastic shortest path is determined as the path which has the largest arrival probability. For path $\Pi$ with node set $N_\Pi$ and arc set $A_\Pi$, following changes in the network imply that path $\Pi$ is the stochastic shortest path; for all $i \in N_\Pi$

- if $j \notin N_\Pi$ and $(j,i) \in A_\Pi$ then $q_{ji}:= 0$ and $q_{jj}:= q_{jj}+q_{ji}$
- if $j \in N_\Pi$ and $(i,j) \notin A_\Pi$ then $q_{ji}:= 0$.

For example, in path $1 \rightarrow 3 \rightarrow 4$ the changes are $q_{23}:=0, q_{22}:=1, q_{14}:=0$. For all of the paths in the example network: path1: $1 \rightarrow 4$, path2: $1 \rightarrow 3 \rightarrow 4$, path3: $1 \rightarrow 2 \rightarrow 3 \rightarrow 4$, Fig. 5 shows path2 to be the stochastic shortest path with the largest arrival probability.

## Numerical results

Some implementations of the proposed method on the networks with different topologies are presented in this section. The instances are directed acyclic networks and there is a path from each node to the destination node. The leaving probabilities of nodes are random numbers produced by the uniform distribution probability. Then, the arrival probability is computed for the established DTMC. All of the experiments are coded in MATLAB R2008a and they are performed on Dell Latitude E5500 (Intel(R) Core(TM) 2 Duo CPU 2.53 GHz, 1 GB memory). To avoid vague demonstration just the stochastic shortest path with the arrival probability computation results are shown by square and circle markers in the figures, respectively; whereas, dashed lines are the results for other paths.

We use two propositions inductively to be sure there will be a path from the source node to the destination node in its initial topology, and the created network is an acyclic network.

### Proposition 1

*If node k is the first node with larger index than source node 1 and*$in{\text{-}}degree(k)=0$, *let*$1 \le l < k$*is an arbitrary node, then by adding arc* (*l*, *k*) *there exists a path from source node 1 to node k*.

### Proposition 2

*If node k is the first node with smaller index than destination nodenand*$out{\text{-}}degree(k)=0$, *let*$k < l \le n$*is an arbitrary node, then by adding arc* (*k*, *l*)*there exists a path from node k to destination noden*.

Network 1 has an arbitrary topology with 8 nodes and 18 arcs and the leaving probabilities of arcs are shown in Table 2. For the established DTMC on network 1, the size of the state space is 47. The absorbing state containing the destination node is accessible by at least two transitions.

**Table 2:** The leaving probabilities of arcs in network 1

(*i*, *j*)	$q_{ij}$	(*i*, *j*)	$q_{ij}$	(*i*, *j*)	$q_{ij}$
(1,2)	0.0115	(2,5)	0.4622	(5,6)	0.1661
(1,3)	0.2059	(2,7)	0.1531	(5,7)	0.3756
(1,5)	0.0080	(3,5)	0.3232	(5,8)	0.1135
(1,6)	0.7532	(3,8)	0.5544	(6,7)	0.3756
(2,3)	0.0877	(4,5)	0.5714	(6,8)	0.4780
(2,4)	0.1900	(4,6)	0.2184	(7,8)	0.8725

**Fig. 6:**
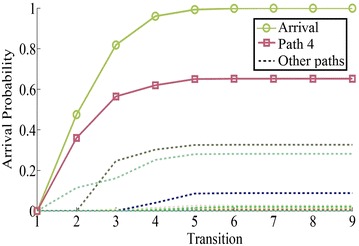
The arrival probability of network 1

As shown in Fig. 6, path 4: $1 \rightarrow 6 \rightarrow 8$ is the stochastic shortest path of network 1 with arrival probability 0.6523 among 27 possible paths.

Network 2 and network 3 are grid networks and the leaving probabilities of their arcs are shown in Table 3. The size of the state space for the established DTMC on network 2 is 76 and for network 3 is 49.

**Table 3:** The leaving probabilities of arcs in network 2 and network 3

Network 2						Network 3			
(*i*, *j*)	$q_{ij}$	(*i*, *j*)	$q_{ij}$	(*i*, *j*)	$q_{ij}$	(*i*, *j*)	$q_{ij}$	(*i*, *j*)	$q_{ij}$
(1,2)	0.0397	(3,5)	0.3229	(5,8)	0.1930	(1,2)	0.9035	(5,6)	0.4611
(1,4)	0.7819	(3,6)	0.4969	(5,9)	0.2432	(1,4)	0.0172	(5,8)	0.3773
(1,5)	0.0883	(4,5)	0.0366	(6,8)	0.3481	(2,3)	0.3828	(6,9)	0.8620
(2,3)	0.4905	(4,7)	0.1349	(6,9)	0.6039	(2,5)	0.5076	(7,8)	0.6601
(2,4)	0.1362	(4,8)	0.7921	(7,8)	0.9531	(3,6)	0.6024	(8,9)	0.6725
(2,5)	0.1199	(5,6)	0.3169	(8,9)	0.7373	(4,5)	0.2695		
(2,6)	0.1360	(5,7)	0.1909			(4,7)	0.7305		

The destination node of network 2 is accessible after at least four transitions, and it is done for network 3 after at least three transitions.

**Fig. 7:**
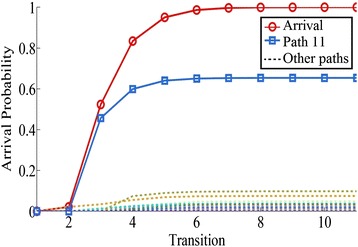
The arrival probability of network 2

**Fig. 8:**
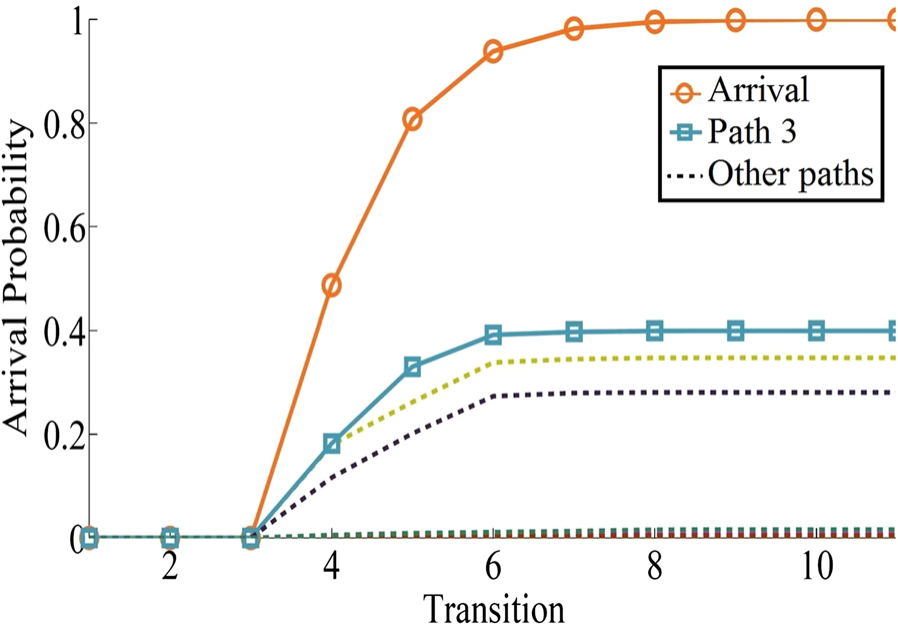
The arrival probability of network 3

As shown in Fig. 7, path 11: $1 \rightarrow 2 \rightarrow 4 \rightarrow 8 \rightarrow 9$ is the stochastic shortest path of network 2 with arrival probability 0.6535 among 33 paths. For network 3, path 3: $1 \rightarrow 2 \rightarrow 5 \rightarrow 6 \rightarrow 9$ is the stochastic shortest path with arrival probability 0.3996 among 6 paths (see Fig. 8).

Network 4 is a complete graph with 9 nodes and 36 arcs and the leaving probabilities are shown in Table 4. The size of the state space for the established DTMC on network 4 is 129.

**Table 4:** The leaving probabilities of arcs in network 4

(*i*, *j*)	$q_{ij}$	(*i*, *j*)	$q_{ij}$	(*i*, *j*)	$q_{ij}$	(*i*, *j*)	$q_{ij}$	(*i*, *j*)	$q_{ij}$
(1,2)	0.0386	(2,3)	0.0540	(3,5)	0.7742	(4,8)	0.0420	(6,9)	0.4366
(1,3)	0.6457	(2,4)	0.0384	(3,6)	0.0322	(4,9)	0.1813	(7,8)	0.4114
(1,4)	0.0272	(2,5)	0.5310	(3,7)	0.0165	(5,6)	0.0977	(7,9)	0.3568
(1,5)	0.0012	(2,6)	0.0338	(3,8)	0.1278	(5,7)	0.1253	(8,9)	0.6908
(1,6)	0.0979	(2,7)	0.1042	(3,9)	0.0180	(5,8)	0.2494		
(1,7)	0.0568	(2,8)	0.0682	(4,5)	0.5820	(5,9)	0.4490		
(1,8)	0.0433	(2,9)	0.1101	(4,6)	0.0358	(6,7)	0.1300		
(1,9)	0.0443	(3,4)	0.0165	(4,7)	0.1545	(6,8)	0.2981		

**Fig. 9:**
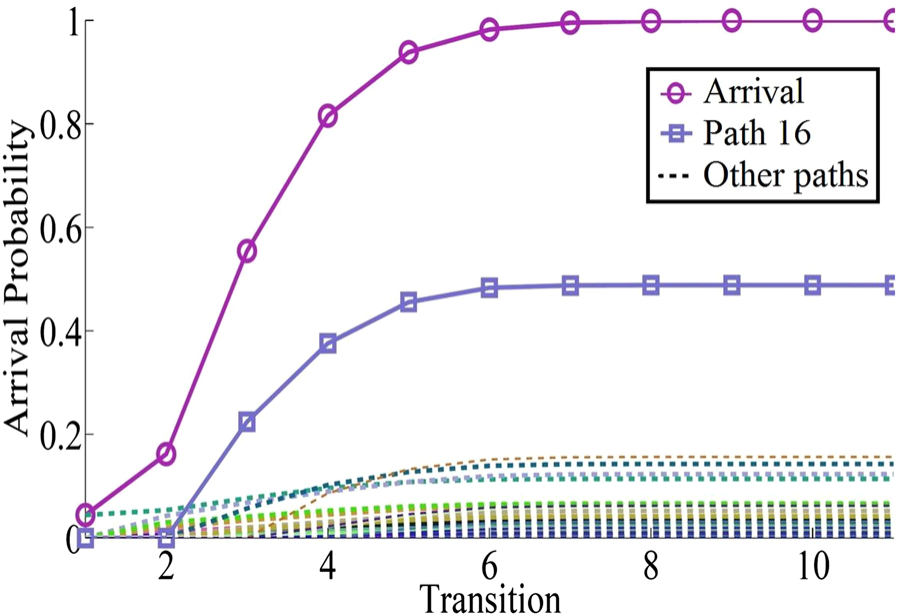
The arrival probability of network 4

The obtained arrival probabilities of network 4 are shown in Fig. 9, and path 16: $1 \rightarrow 3 \rightarrow 5 \rightarrow 9$ is the stochastic shortest path with arrival probability 0.4882 among 128 possible paths.

The obtained arrival probability in a network determines the general situation of the network to transmit flow from a source node toward a destination node (Shirdel and Abdolhosseinzadeh 2016); however, the presented method precisely determines the path with the largest probability amongst all paths.

## Conclusions

The arrival probability from a given source node to a given destination node was computed according to the probability of transition from the initial state to the absorbing state by multi-step transition probability of the established discrete time Markov chain in the original network. The proposed method to obtain the arrival probability determines that the destination node is accessible for the first time. The stochastic shortest path was separately determined which has the largest arrival probability value. So, this method can be applied to rank paths of a network by considering their obtained arrival probabilities. Also, the proposed method evaluates the reliability of connections in the networks. So, it can be used in the shortest path problem with recourse, where locally should be decided which path is selected to traverse. The discrete nature of the proposed model could apply meta-heuristic methods to reduce the computations. Also, the proposed method can be used for the stochastic problems as a policy evaluation index.
